# Editorial: Complex network dynamics in consciousness

**DOI:** 10.3389/fncom.2023.1310392

**Published:** 2023-11-01

**Authors:** Francisco J. Esteban, Antonio Ibáñez-Molina, Sergio Iglesias-Parro, Juan Ruiz de Miras, Fernando Soler-Toscano

**Affiliations:** ^1^Experimental Biology Department, University of Jaén, Jaén, Spain; ^2^Psychology Department, University of Jaén, Jaén, Spain; ^3^Software Engineering Department, Research Center for Information and Communication Technologies (CITIC-UGR), University of Granada, Granada, Spain; ^4^Philosophy, Logic and Philosophy of Science Department, Sevilla University, Sevilla, Spain

**Keywords:** dynamic system, consciousness, complexity, network, brain

## Introduction

Consciousness is a complex and fascinating phenomenon that has been studied by philosophers and scientists for centuries (Block, 1993; Chalmers, 1995; Damasio, 1995; Appleby et al., 1996; Seth, 2018). It is the subjective experience of awareness, and it is essential for our ability to interact with the world around us. While we have made significant progress in understanding the neural and cognitive underpinnings of consciousness, there is still much that we do not know (Crick and Koch, 2003; Tononi and Koch, 2008; Birch et al., 2020).

One promising approach to understanding consciousness is to study it as a complex network phenomenon because the brain is a complex network of neurons and glial cells that are interconnected in intricate ways (Bullmore and Sporns, 2009; Rubinov and Sporns, 2010; Dehaene, 2014; Avena-Koenigsberger et al., 2018). The dynamics of these networks are thought to play a key role in consciousness.

**Figure 1:**
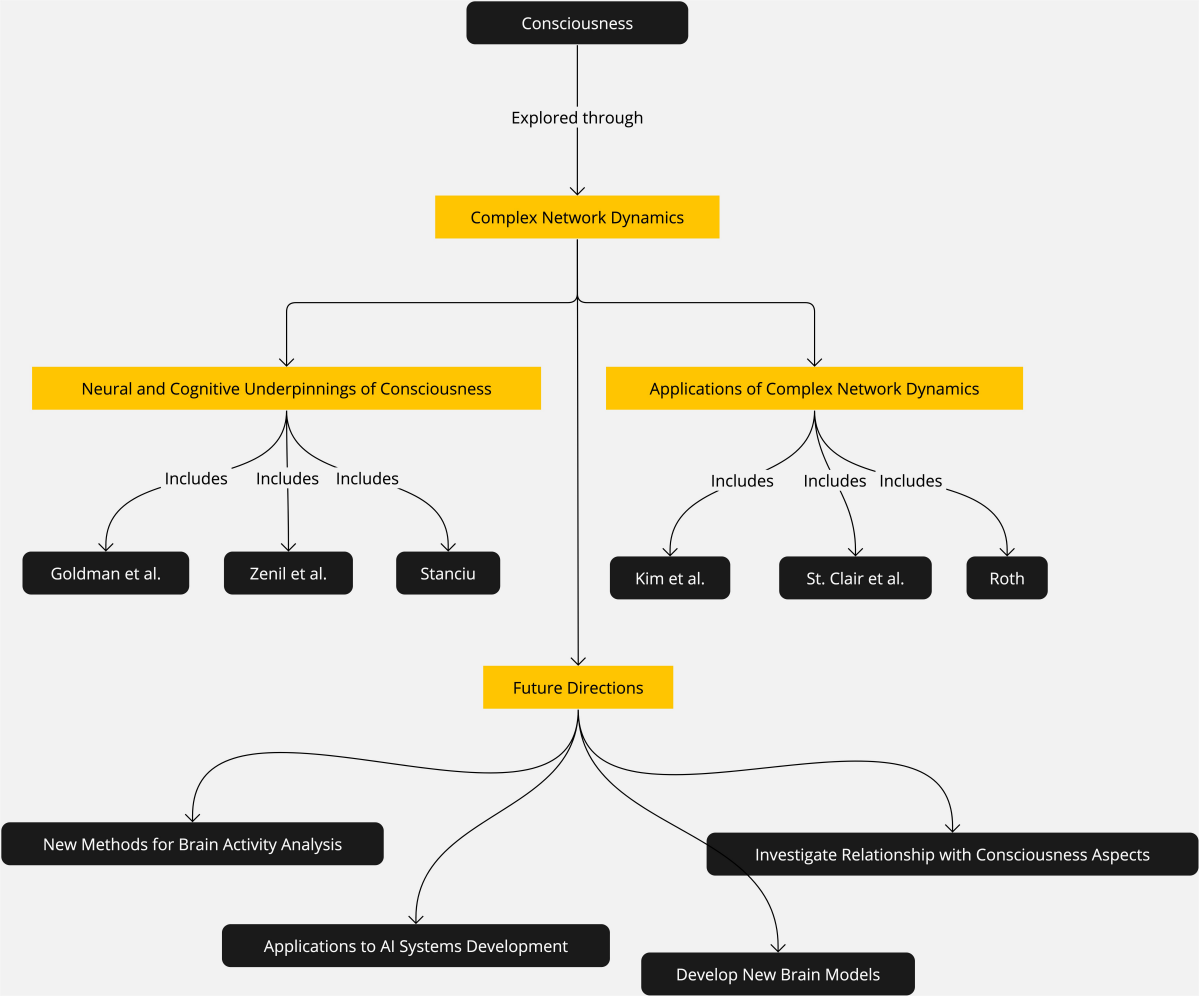
Summary of what has been the Research Topic complex network dynamics in consciousness.

In this Research Topic (see Figure 1), we have gathered a collection of articles that explore the complex network dynamics of consciousness from different perspectives, including the role of small-world and scale-free networks, synchronization and coherence, network plasticity, and the relationship between consciousness and information processing.

Taken together, the articles presented here provide new insights into the complex network dynamics of consciousness and how these dynamics may contribute to our subjective experience. These articles can be grouped into the next two sections.

## Neural and cognitive underpinnings of consciousness

The articles by Goldman et al., Zenil et al., and Stanciu all focus on the neural and cognitive underpinnings of consciousness. Goldman et al. develop a comprehensive neural simulation model that accounts for the properties of empirically observed spontaneous and stimulus-evoked dynamics in space, time, phase, and frequency domains.

Zenil et al. develop a toolbox to objectively characterize the intrinsic complexity of behavioral patterns resulting from human or animal decisions. Stanciu explores the relationship between the new approach in cognitive science known as “4E cognition” and Aristotle's views on mind and body.

These three articles provide complementary perspectives on the neural and cognitive underpinnings of consciousness. Goldman et al.'s model provides a valuable tool for studying the neural dynamics of consciousness and other brain states. Zenil et al.'s toolbox could be used to investigate the role of behavioral complexity in consciousness and other cognitive processes. Stanciu's analysis of 4E cognition and Aristotle's views on mind and body offers a new perspective on the mind-body problem and the relationship between consciousness and cognition.

## Applications of complex network dynamics

The articles by Kim et al., St. Clair et al., and Roth all explore the potential applications of complex network dynamics to the development of new artificial intelligence (AI) systems and the treatment of consciousness-related disorders. Kim et al. develop a large-scale brain network model to study the effects of explosive synchronization (ES) on hypersensitivity in the Fibromyalgia (FM) brain. St. Clair et al. develop a novel learning model, which they term the “Recommendation Architecture (RA) Model,” which uses both consequence feedback and non-consequence feedback. Roth proposes a quantum-like description of neuron activity using Quantum Like-Cellular Automaton (QLCA) concepts.

These three articles offer new and innovative approaches to using complex network dynamics to develop new AI systems and to treat consciousness-related disorders. Kim et al.'s model suggests that network modulation could be used to reduce hypersensitivity in FM patients. St. Clair et al.'s RA model could be used to develop more efficient AI systems. Roth's quantum-like description of neuron activity provides a new perspective on neuron activity and its potential relationship to quantum mechanics.

## Future directions

The research presented in the previous two sections provides new insights into the complex network dynamics of consciousness and how these dynamics may contribute to our subjective experience. This research highlights the importance of future research in this area to deepen our understanding of consciousness and its relationship to the brain.

Thus, the articles in this Research Topic provide a valuable foundation for future research on the complex network dynamics of consciousness. In this sense, one important area of future research is to develop new and improved models of the brain as a complex network. These models should be able to account for the rich and diverse dynamics of brain activity, as well as the interactions between different brain regions. This research could lead to a better understanding of how the brain gives rise to consciousness.

Another important area of future research is to investigate the relationship between complex network dynamics and different aspects of consciousness, such as attention, perception, memory, and decision-making. This research could lead to a better understanding of the neural basis of consciousness and to the development of new treatments for consciousness-related disorders such as coma, vegetative/unresponsive wakefulness syndrome and minimally conscious states.

Future research should also explore the potential applications of complex network dynamics to the development of new AI systems. And also new methods for recording and analyzing brain activity, as well as new computational models and algorithms for simulating and analyzing complex networks.

## Conclusion

The study of complex network dynamics in consciousness is a rapidly growing field with the potential to revolutionize our understanding of this fundamental phenomenon. The research discussed in this Research Topic provides a valuable foundation for future work in this area, and it points the way to many exciting directions for future research. The study of complex network dynamics in consciousness is a rapidly growing field with the potential to revolutionize our understanding of this fundamental phenomenon and to lead to new applications in AI and clinical practice.

## Author contributions

FE: Writing – original draft. AI-M: Writing – review & editing. SI-P: Writing – review & editing. JR: Writing – review & editing. FS-T: Writing – review & editing.
